# Antenatal Imaging and Neonatal Outcome in Infants with Congenital Cytomegalovirus Infection: The Effect of Valaciclovir

**DOI:** 10.3390/jcm15020809

**Published:** 2026-01-19

**Authors:** Francesca Arcieri, Adele Vasta, Sara Sorrenti, Gregorio Volpe, Valentina D’Ambrosio, Daniele Di Mascio, Fabio Natale, Lucia Manganaro, Giuseppina Liuzzi, Maria Caterina Corigliano, Sara Bertolini, Stella Borza, Carla Camerino, Giuseppe Rizzo, Antonella Giancotti

**Affiliations:** 1 Department of Maternal and Child Health and Urological Sciences, Sapienza University of Rome, 00161 Rome, Italy; francesca.arcieri@uniroma1.it (F.A.); adele.vasta@uniroma1.it (A.V.); sara.sorrenti@uniroma1.it (S.S.); gregorio.volpe@uniroma1.it (G.V.); daniele.dimascio@uniroma1.it (D.D.M.); stella.borza@uniroma1.it (S.B.); carla.camerino@uniroma1.it (C.C.); giuseppe.rizzo@uniroma1.it (G.R.); 2 Department of Gynecology, Obstetrics and Urological Sciences, Azienda Ospedaliero-Universitaria Policlinico Umberto I, 00161 Rome, Italy; dr.valentina.dambrosio@gmail.com (V.D.); fab.natale@libero.it (F.N.); m.corigliano@policlinicoumberto1.it (M.C.C.); 3 Department of Radiological, Oncological and Pathological Sciences, Sapienza University of Rome, 00161 Rome, Italy; lucia.manganaro@uniroma1.it; 4 Maternal–Fetal Infection Prevention Service, National Institute for Infectious Diseases Lazzaro Spallanzani IRCCS, 00149 Rome, Italy; giuseppina.liuzzi@inmi.it; 5 Faculty of Medicine and Dentistry, Sapienza University of Rome, 00161 Rome, Italy; sara.bertolini17@gmail.com

**Keywords:** congenital cytomegalovirus infection, CMV, Cytomegalovirus, primary infection, pregnancy, symptomatic infection, valacyclovir, amniocentesis

## Abstract

**Background:** Congenital cytomegalovirus (cCMV) infection is a leading cause of neonatal morbidity. This retrospective study aimed to evaluate the efficacy of valacyclovir in reducing vertical transmission after primary maternal CMV infection and to assess the diagnostic performance of amniocentesis and prenatal imaging. **Methods:** Eighty-two pregnant women with confirmed primary CMV infection were included. Maternal CMV serology and viral DNA were assessed in blood and urine, with standardized prenatal care including serial ultrasound examinations and fetal MRI when indicated. Amniocentesis was offered to confirm fetal infection. Valacyclovir (8 g/day) was administered before 24 weeks’ gestation, and neonatal infection was diagnosed by CMV DNA detection in urine at birth. Statistical analyses were performed using SPSS version 27.0. **Results:** Most infections (62.2%) were diagnosed in the first trimester. Valacyclovir was administered in 97.6% of cases, and amniocentesis was performed in 81.7%, with CMV DNA detected in 19.4%. Among 74 live births, 23% of neonates were CMV-positive and 6.8% symptomatic. Seven infected neonates had negative amniocentesis (false-negative rate, 13.2%). Prenatal ultrasound and MRI failed to detect abnormalities in symptomatic cases. **Conclusions:** Valacyclovir may reduce, but does not eliminate, the risk of cCMV transmission. Negative amniocentesis does not fully exclude fetal infection, highlighting postnatal follow-up.

## 1. Introduction

**Figure 1:**
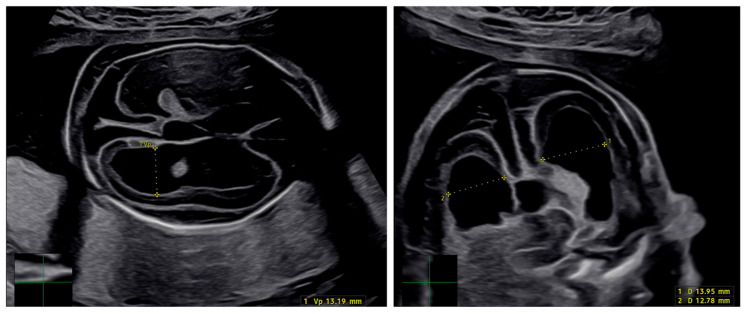
Prenatal ultrasound at 20w + 2d of gestation showing moderate bilateral ventriculomegaly in a fetus with confirmed congenital cytomegalovirus (CMV) infection.

Congenital cytomegalovirus (cCMV) infection is the most common congenital infection worldwide, with a global prevalence of 1 to 156 infants [1], and remains the leading non-genetic cause of sensorineural hearing loss [2,3]. Prenatal diagnosis is feasible and typically performed following a positive first-trimester serological screening or the detection of ultrasound findings suggestive of viral infection. cCMV can result from either a primary or non-primary maternal infection, with vertical transmission occurring via transplacental passage of virus-infected cells. The incidence of primary maternal infection is approximately 1–2%, with an overall rate of vertical transmission of 32% [4,5]. The prevalence of non-primary infections is less well defined; however, a three-year study in the United States reported an annual incidence of 10% among young women of seropositive pregnant women [4]. Most affected infants are asymptomatic at birth, but approximately 10–15% present with clinical signs such as petechiae or purpura, low birth weight, hepatosplenomegaly, microcephaly, seizures, and retinitis [5]. Numerous studies have demonstrated that the extent of fetal and neonatal damage—particularly severe neurological impairment—correlates with the gestational age at which vertical transmission occurs, with the highest risk associated with infections acquired during the periconceptional period and first trimester [6]. Nevertheless, mild neurological sequelae and sensorineural hearing loss may also occur following infections acquired in the second or third trimester [7,8]. A meta-analysis found that while long-term sequelae are more common in infants with clinically evident disease at birth, approximately 13.5% of asymptomatic infants also develop long-term complications attributable to cCMV [8]. Prenatal ultrasound findings suggestive of congenital CMV infection include ventriculomegaly (as shown in Figure 1), periventricular echogenicity and calcifications, microcephaly, intracranial cysts, echogenic bowel, hepatosplenomegaly, ascites, growth restriction, and placental thickening. Fetal magnetic resonance imaging may further detect cortical malformations, white matter abnormalities, delayed sulcation, and migrational disorders, improving the assessment of central nervous system involvement, although its prognostic accuracy remains limited, particularly in early or late gestational transmission. Despite these imaging capabilities, prenatal ultrasound and fetal MRI have limited prognostic accuracy, and congenital CMV infection may occur even in the absence of detectable antenatal abnormalities [9].

Valacyclovir (VCV), an antiviral agent used to treat herpesvirus infections, has recently been proposed as a therapeutic option for pregnant women with primary CMV infection. Recent clinical studies have increasingly investigated the role of antiviral therapy in reducing the risk of congenital cytomegalovirus (CMV) transmission. In particular, a randomized controlled trial by Shahar-Nissan et al. (2020) showed that high-dose Valacyclovir (8 g/day) significantly decreased vertical transmission when administered to women with primary CMV infection during the first trimester, reducing transmission rates from 30% to 11% compared to placebo [10]. More recently, a systematic review and meta-analysis by D’Antonio et al. (2023) confirmed the effectiveness of prenatal Valacyclovir in reducing congenital CMV infection, especially when maternal infection occurs early in gestation [11]. Based on the most recent evidence [10,12], in December 2020 Valacyclovir was officially included among the reimbursable drugs by the Italian National Health System. This study aims to assess the efficacy of Valacyclovir treatment during pregnancy by evaluating neonatal outcomes in patients who received antiviral therapy.

## 2. Materials and Methods

### 2.1. Study Design and Patient Selection

This retrospective study was conducted by collecting data from all pregnancies with confirmed primary CMV infection referred to the Prenatal Diagnosis Centre of the Department of Maternal and Child Health and Urological Sciences at Policlinico Umberto I, Sapienza University of Rome, between January 2022 and March 2025. This is a tertiary referral center for maternal and fetal infections, managing an average of approximately 35 pregnancies per year with suspected or confirmed primary CMV infection.

Inclusion criteria included women aged over 18 years with a diagnosis of primary CMV infection acquired during the periconceptional period, first, second, or third trimester of pregnancy. The diagnosis was based on the following serological and molecular findings: positive CMV IgM and negative or low-positive IgG with a low IgG avidity index during pregnancy; positive CMV IgG and IgM with an intermediate IgG avidity index, associated with CMV DNA positivity in at least one bodily fluid (blood, saliva, or urine) during pregnancy. Exclusion criteria included: Non primary infection (reinfection or reactivation) that presented high avidity. Patients with primary infection decided for termination of pregnancy.

### 2.2. Methods

Women who were diagnosed with primary CMV infection received prenatal follow up care during pregnancy adhering to a structured protocol that included:

- Collection of accurate medical history, performance of general objective examination and obstetrical examination with particular attention on identifying flu-like symptoms (fever, asthenia, myalgia).
- In the preliminary consultation, assessment of the maternal serological profile to identify whether it was a primary infection or a possible reactivation.
- Reassessing serological tests (IgG, IgM), checking avidity levels and examining the viral genome present in blood and urine specimens.
- Clinical evaluation by an infectious disease consultant.
- Scheduled ultrasound scans to identify signs of fetal infection.

Counselling on CMV infection and current diagnostic and therapeutic possibilities was offered.

Once the primary infection has been confirmed, invasive prenatal diagnosis was offered and scheduled by amniocentesis 6–8 weeks after CMV seroconversion and no earlier than the 20th week of gestation, for the detection of the viral genome in the amniotic fluid using a standardized quantitative Real Time PCR system. The patients were informed of the risks associated with this procedure. Amniocentesis was always performed under ultrasound guidance using 21-gauge needle, avoiding the placenta and umbilical cord insertion.

All patients underwent systematic evaluations by obstetricians during their pregnancies utilizing both transabdominal and transvaginal ultrasound scans every 4–6 weeks. This approach aimed to identify ultrasound markers of congenital infection, which would help in the prediction of the severity of potential neonatal complication.

The ultrasound examinations were performed according to the guidelines of the Italian Society of Obstetric and Gynecological Sonography (SIEOG).

Patients with primary seroconversion were offered fetal Magnetic Resonance Imaging (Fetal MRI) for a more detailed study of the Central Nervous System. Fetal MRI was performed with T2 HASTE, static and dynamic TRUEFISP, T1 VIBE, T1 flash 2D sequences and with diffusion technique, according to multiple scan planes, under basal conditions.

Since 16 December 2020, following the introduction by the Italian drug agency of Valacyclovir in the list of medicines that can be dispensed at full charge of the National Health Service, patients with primary CMV infection within the 24th week of gestation have been offered high dose Valacyclovir therapy (2 g every 6 h, for a total of 8 g/day).

Data regarding perinatal and neonatal outcomes were collected: These included gestational age at birth, the presence of the virus in blood and urine, the presence of anomalies at birth attributable to CMV infection with particular attention to brain lesions and signs of hearing loss obtained by acoustic evoked potentials (ABR). Follow-up information concerning children, who were not delivered in our Unit, was obtained by phone interview. Neonatal confirmed infection was defined as the presence of CMV DNA in the neonates’ urine.

### 2.3. Data Management

Personal data has been managed in agreement with local personal data protection rules. All personal identification information has been deleted to anonymize data sets.

### 2.4. Statistical Analysis

Data was analyzed using SPSS (v27.0) package. Continuous variables were expressed in mean and standard deviations; categorical data were presented as absolute numbers and frequences (%). Normality was evaluated using the Shapiro–Wilk test. Normally distributed variables were analyzed using the t-Student test, whereas non normally distributed variables were evaluated with Mann–Whitney test. Chi square test or Fisher’s exact test were used to evaluate categorical variables, as appropriate. The value of *p* < 0.05 was considered statistically significant. Univariate logistic regression analysis was also performed to evaluate the association between risk factors and the occurrence of confirmed neonatal infection and symptomatic neonatal infection. In addition, a diagnostic test accuracy analysis was performed to evaluate the performance of prenatal testing in predicting cases of neonatal infection.

## 3. Results

The search identified 104 pregnant patients diagnosed with cytomegalovirus (CMV) infection. Among these, 82 were cases of primary infection, while 22 were excluded because they involved reinfection or reactivation.

Out of the 82 patients with primary CMV infection, 50 (62.5%) were diagnosed during the first trimester, 31 (38.7%) during the second trimester, and only one case (1.2%) occurred in the third trimester. Maternal characteristics of the study cohort are displayed in Table 1.

Of the 82 patients considered in this study, 80 received Valacyclovir treatment: of the remaining two patients, one decided to interrupt the pregnancy and the other acquired the infection in the third trimester; therefore, treatment was not indicated [13].

A total of 67 out of 82 patients (81%) underwent amniocentesis during pregnancy. The remaining 15 women refused the related diagnostic procedure. The presence of CMV DNA in amniotic fluid was detected by real-time PCR in 13 patients (19.4%): 69.23% in the first trimester group (9/13) 30.77% in the second trimester group (4/13). The average viral load in the amniotic fluid for patients who tested positive was 1,681,782 ± 3,271,324 copies.

Ultrasound anomalies were identified in 12 patients, including 12 cases of hyperechogenic bowel (14.6%). In addition, 63 out of 82 patients underwent fetal MRI, which detected abnormalities in 3 cases: one with ventriculomegaly, one with an abdominal cyst, and a third featuring a horseshoe kidney.

**Figure 2:**
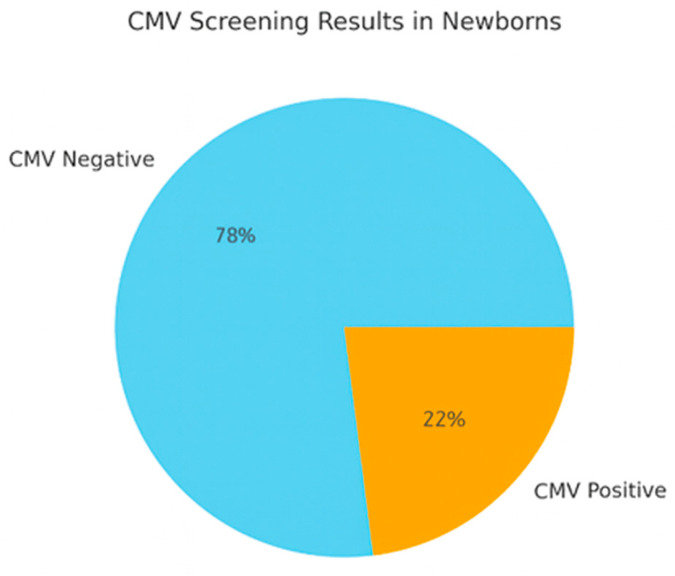
This chart shows the neonatal CMV outcomes among the 73 mothers treated with Valacyclovir: 78% of newborns tested negative for CMV at birth and 22% tested positive, indicating that Valacyclovir may reduce—but not entirely prevent—congenital CMV transmission.

Among the 82 patients with primary infection, 8 patients (9.8%) opted for termination of pregnancy: 7 (20%) presented a positive amniotic fluid, and one had Myocardial hypertrophy and high avidity with negative amniocentesis. 74 (90.2%) chose to continue the pregnancy. Of the 74 pregnancies that resulted in live births, 73 mothers had received Valacyclovir treatment. Among all newborns, 57 (77%) tested negative for CMV, whereas 17 (22.9%) showed positive results at CMV screening. Of the positive cases, 5 (29.4%) were diagnosed with positive amniocentesis; 7 cases (41.2%) had negative amniocentesis; 5 other cases did not have invasive testing. One of the congenital CMV cases was not treated with Valacyclovir prenatally; all the other cases were treated (Figure 2).

The diagnostic test accuracy analysis showed that a positive amniocentesis only had 41.7% (95% CI 17.5–69.1) sensitivity in identifying cases of neonatal CMV infection (Table 2); on the other hand, specificity was high (97.9%, 95% CI 91.0–99.9), as well as had positive and negative predictive values (83.3%, 95% CI 44.6–99.0 and 86.8, 95% CI 76.0–94.1, respectively). Ultrasound findings in the II trimester showed low sensitivity (12.5%, 95% CI 2.2–33.8) and specificity (79.6, 95% CI 67.7–88.9) in predicting neonatal infection. The third trimester scan, instead, showed slightly higher, yet low, sensitivity (23.5%, 95% CI 8.0–46.5) and positive and negative predictive values (36.4%, 95% CI 13.0–65.4 and 77.2%, 95% CI 65.3–86.7, respectively); specificity for US findings at this gestation was moderate (86.3%, 95% CI 75.1–93.9). MRI prenatal findings showed moderate negative predictive value (75.0%, 95% CI 63.1–84.8).

The univariate regression analysis of predictors of neonatal confirmed infection showed that positive amniocentesis, regardless of the low sensitivity, was strongly associated with the outcome (OR 32.857, 95% CI 3.329–324.316). Similarly, GA at end of treatment with Valacyclovir (OR 1.232, 95% CI 1.077–1.411) and duration of treatment in weeks (OR 1.135, 95% CI 1.011–1.274), showed a positive association with the occurrence of neonatal infection. This was consistent with the 69.6% reduction in rates of neonatal infections found in case of interruption of treatment < 25 weeks (OR 0.304, 95% CI 0.097–0.953). The latter group, in fact, mostly comprehended cases of negative amniocentesis that stopped the treatment. No significant association was observed between the GA at start of treatment or findings at prenatal echo and neonatal infection (Table 3).

Regarding the prediction of symptomatic neonatal infection, no predictor showed significance association with the outcome (Table 3).

**Figure 3:**
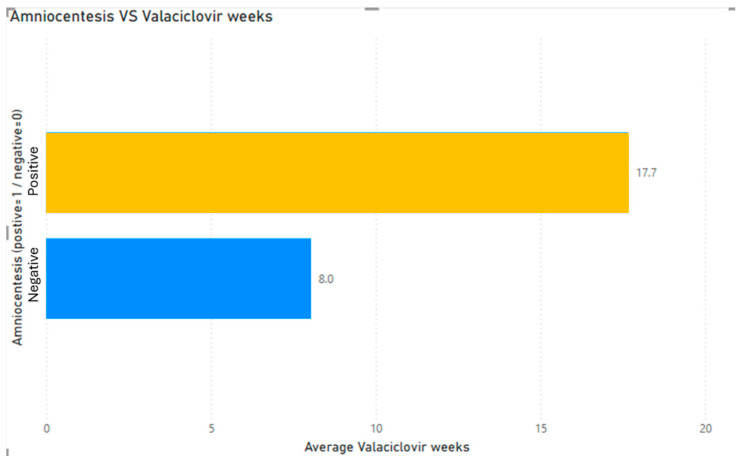
Duration of treatment with Valacyclovir.

The mean duration of Valacyclovir treatment among women with a positive amniocentesis was 17.7 ± 5.7 (maximum 27 weeks, minimum 10) whereas those with a negative result had a men treatment duration of only 8 ± 3.4 weeks (minimum 2 maximum 16, with Valacyclovir being discontinued immediately after the negative amniocentesis) (Figure 3).

**Figure 4:**
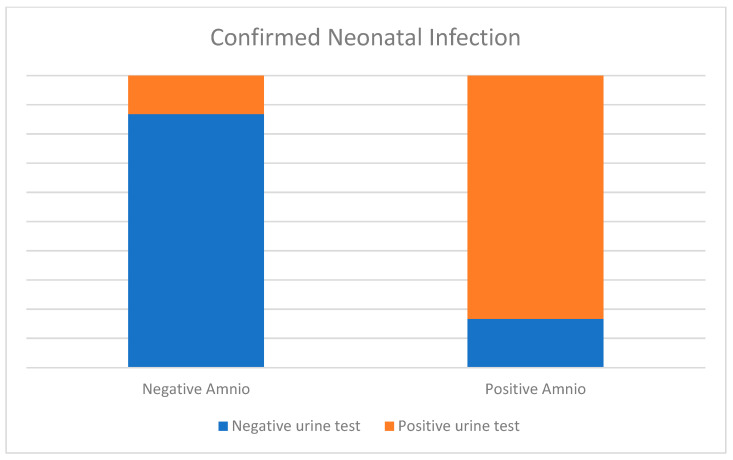
Comparison of prenatal amniocentesis results with postnatal urine test results. Among infants with negative amniocentesis (n = 53), 89.8% had a negative urine test at birth. Of those with positive amniocentesis (n = 6), 83.3% had a positive urine test.

Among the 73 newborns, 59/73 (80.8%) patients choose to do amniocentesis: 53/59 (89.8%) was negative and 6/59 (10.2%) was positive: comparing to urine test results at birth we see that about negatives, 46/53 (86.8%) infants with negative amniocentesis also tested negative at birth, and 7/53 (13.2%) tested positive and about positive, 5/6 infants (83.3%) tested positive at birth, and only 1/6 (16.6%) tested negative; 3 of 5 (60%) positive cases were symptomatic patients (Figure 4).

Neonatal CMV infection was confirmed at birth in 16 of the 73 newborns (22%) by detecting CMV DNA in their urine. Among the 16 infants with confirmed congenital CMV infection, 10 (62.5%) were asymptomatic and 6 (37.5%) were symptomatic. Symptomatic cases were defined as those presenting with neurological abnormalities on imaging, chorioretinitis, thrombocytopenia, hepatosplenomegaly with altered organ function markers in blood tests, or hearing loss at birth.

Postnatal cerebral ultrasound revealed abnormalities in 3 of the 6 symptomatic infants: all three showed periventricular calcifications, with one case also presenting cerebral vasculopathy. Another infant displayed vasculopathy along with germinal matrix cysts. Post-natal MRI was performed in 2 symptomatic infants, both of which showed abnormalities. One MRI revealed isolated periventricular calcifications, while the other showed both periventricular calcifications and polymicrogyria. The last one was the only preterm infant and the one with the most severe neurological symptoms, requiring postnatal treatment with Valganciclovir. Notably, none of the symptomatic infants had ultrasound or MRI abnormalities identified prenatally (Table 4). 

Additional findings in infants who tested positive at birth included: Abnormal transient otoacoustic emissions (TOAEs): 2 cases, Fetal anemia: 2 cases, Elevated transaminases: 1 case, Elevated direct bilirubin levels: 4 cases None of the infants presented with hepatosplenomegaly, chorioretinitis, or thrombocytopenia at birth. Importantly, no significant adverse effects related to Valacyclovir treatment were observed in any of the patients included in this study.

## 4. Discussion

The management of primary CMV infection during pregnancy remains clinically challenging due to the variability in fetal transmission, the limited sensitivity of available diagnostic tools, and the heterogeneity of neonatal outcomes. Within this context, our study provides new insights into the diagnostic value of amniocentesis, the prognostic limitations of prenatal imaging, and the effectiveness of Valacyclovir treatment.

One of the key findings concerns the role of amniocentesis. Although only 10% of procedures yielded a positive result, amniocentesis positivity showed high specificity and strong predictive value for congenital CMV infection (PPV 95% CI 0.8333 (0.446–0.990), emerging as the most powerful independent predictor of neonatal infection. (OR 32.9; 95% CI: 3.3–324.3; *p* = 0.003) However, the identification of seven infected infants despite a prior negative amniocentesis highlights the test’s limited sensitivity, a phenomenon previously described in the literature [14,15,16]. Possible explanations include late transplacental transmission, false-negative results due to suboptimal timing, or viral replication following discontinuation of Valacyclovir therapy, as suggested by recent reports. These observations align with the meta-analysis by Chatzakis et al. [6] which reported an 8% false-negative rate but found no severe neonatal outcomes among cases with negative amniocentesis. Prenatal counseling must therefore remain balanced: a negative amniocentesis substantially reduces—yet does not eliminate—the risk of clinically significant disease.

Consistent with this interpretation, the recent review by D’Alberti et al. [17] further highlights that amniocentesis—while the diagnostic gold standard—may still fail to detect fetal infection in selected circumstances, particularly when transplacental transmission occurs later in gestation or when viral load at the time of sampling is low. Consistent with previous evidence, no therapeutic abortions occurred in our cohort when amniocentesis was negative and imaging was reassuring, underscoring its value in risk stratification.

Moreover, our analysis highlights the substantial limitations of prenatal imaging in predicting neonatal outcomes. Ultrasound abnormalities were observed in only a minority of cases (14%), and none of the symptomatic infants in our cohort had abnormal prenatal ultrasound or fetal MRI findings regarding CMV congenital infection. Relevant abnormalities were detected exclusively postnatally—most notably periventricular calcifications and vasculopathy—and, in the two symptomatic cases who underwent postnatal MRI, additional findings such as calcifications and polymicrogyria were detected. Both second- and third-trimester ultrasound demonstrated low sensitivity (12.5% and 23.5%, respectively) and limited positive predictive value (15.4% and 36.4%), particularly when abnormalities were subtle or non-specific. Likewise, fetal MRI, although showing a negative predictive value of 75%, was not able to reliably exclude congenital infection—a limitation also noted by Di Mascio et al. [9], who reported that MRI may detect abnormalities invisible to ultrasound but remains influenced by timing of transmission and fetal viral load. Consistent with this observation, the multicenter study by Mappa et al. [18] demonstrated that even fetuses with normal prenatal imaging at the time of diagnosis may subsequently develop disease-related findings: 18.3% showed structural abnormalities at follow-up ultrasound or MRI, and an additional 11.9% were diagnosed only postnatally. Notably, 15.5% of infants were symptomatic at birth despite reassuring early imaging, further underscoring the inability of prenatal assessment alone to reliably rule out clinically significant congenital CMV. The discrepancy between prenatal imaging and neonatal clinical presentation is further exemplified by echogenic bowel, the most frequent ultrasound anomaly in our cohort, which did not correlate with symptomatic infection. Echoing observations by Vena et al. and De Coninck [19,20], isolated echogenic bowel lacks prognostic reliability without virological confirmation, as it may appear in both infected and uninfected fetuses.

These limitations are reflected in current clinical guidelines. While the ECCI [4] recommends routine antenatal care following a negative amniocentesis, the RCOG [21] advocates serial ultrasound surveillance, including fetal MRI, in confirmed or possible infections. However, as highlighted in the guideline review by Sorrenti et al. [22], no international consensus exists regarding the systematic use of advanced fetal imaging, reflecting insufficient evidence on its true prognostic value and raising concern for potential underestimation of clinically relevant yet silent infections.

Another relevant finding from our study concerns the association between antiviral treatment timing and neonatal infection. Discontinuation of therapy before 25 weeks was associated with a reduced risk of congenital infection (OR 0.30; *p* = 0.041), supporting the hypothesis that pregnancies with negative amniocentesis—in which treatment was typically stopped—are more likely to have a favorable outcome. By contrast, none of the evaluated prenatal variables, including amniocentesis positivity (OR 3.75; *p* = 0.286), prenatal ultrasound findings, or treatment timing, were significant predictors of symptomatic infection at birth, underscoring the difficulty of accurately stratifying neonatal clinical severity using prenatal markers alone.

Valacyclovir was well tolerated in our cohort, with no significant maternal or fetal adverse events observed. Reported side effects in the literature are generally mild and include gastrointestinal symptoms, headache, and transient renal function alterations, particularly at high doses, confirming the favorable safety profile of this antiviral during pregnancy [11].

In addition to antiviral therapy, behavioral preventive strategies may play a relevant role in reducing maternal exposure to CMV, particularly during periods of increased viral circulation. Although CMV is not a respiratory virus, transmission frequently occurs through close contact with infected bodily fluids, especially saliva and urine, which are common in childcare and household settings. Simple hygienic measures such as frequent handwashing, avoidance of contact with saliva (e.g., sharing utensils or kissing young children on the mouth), and careful handling of diapers have been shown to reduce CMV seroconversion rates. Interestingly, evidence from the COVID-19 pandemic suggests that increased awareness and adoption of preventive behaviors during pregnancy may significantly reduce the risk of viral exposure. Stampini et al. [23] demonstrated a significantly lower SARS-CoV-2 seroprevalence among pregnant women compared to non-pregnant women, hypothesizing that a more cautious lifestyle—including hygiene measures and physical distancing—may have contributed to this finding. Although derived from a different viral context, these observations support the concept that targeted counseling and behavioral interventions during pregnancy may be beneficial also for CMV prevention, particularly in high-risk seasons or settings.

Maternal viral load also demonstrated limited utility as a predictive marker. Although higher in mothers of infected infants, differences did not reach statistical significance, reaffirming evidence from Leruez-Ville [6] that isolated maternal viremia is not a reliable indicator of fetal infection. In contrast, neonatal viral load has demonstrated strong prognostic relevance, with levels below 3 log_10_ IU/mL showing a 100% negative predictive value for long-term sequelae, as reported by Fourgeaud et al. [24].

This study is strengthened by its comprehensive diagnostic approach, combining amniocentesis, prenatal ultrasound, and fetal magnetic resonance imaging, which allowed an in-depth evaluation of congenital CMV transmission and neonatal outcomes. The analysis of false-negative amniocentesis results further highlights the diagnostic challenges of congenital CMV and reinforces the importance of postnatal surveillance.

However, the relatively small sample size may limit the generalizability of the findings. Moreover, the persistence of congenital infection and symptomatic cases despite antiviral therapy confirms that valacyclovir is only partially effective, underscoring the need for larger studies and further research to improve preventive and diagnostic strategies.

## 5. Conclusions

Valaciclovir is confirmed as a safe and potentially effective therapy for reducing vertical transmission in maternal primary infection. However, negative amniocentesis does not entirely exclude fetal infection, as viral replication may reactivate after treatment discontinuation. Prenatal imaging provides limited predictive value. A multidisciplinary approach and long-term neonatal follow-up remain essential to identify and manage symptomatic cases that may escape prenatal diagnosis.

## Figures and Tables

**Table 1:** Prenatal General Characteristics.

Characteristic	Mean (SD) or *n* (%)
Mean maternal age	31.6 years ± 6.43
Trimester of primary infection diagnosis	1st trimester: 50 (62.2%) 2nd trimester: 30 (36.6%) 3rd trimester: 1 (1.2%)
Received Valacyclovir treatment	80/80 patients (100%)
Mean duration of Valacyclovir treatment	8.7 weeks ± 4.71
Patients who underwent amniocentesis	67/82 (81.7%)
Positive amniocentesis (CMV DNA in amniotic fluid)	13/67 (19.4%)
Mean viral load in amniotic fluid (positive cases)	1,681,782 ± 3,271,324 copies
Mean CMV DNA in maternal urine	7,569,843 ± 10,704,833 copies
Mean CMV DNA in maternal blood	11,400 ± 6760 copies
Fetal ultrasound anomalies	12 cases (14.6%): 12 hyperechogenic bowel
Fetal MRIs performed	63/82 patients (76.8%)
Anomalies detected on fetal MRI	3 cases: one with ventriculomegaly (not confirmed at birth), one with an abdominal cyst one with a horseshoe kidney.

**Table 2:** Diagnostic test accuracy for neonatal infection.

	Sensitivity (95% CI)	Specificity (95% CI)	Positive Predictive Value (95% CI)	Negative Predictive Value (95% CI)
Positive amniocentesis	0.417 (0.175–0.691)	0.979 (0.910–0.999)	0.833 (0.446–0.990)	0.868 (0.760–0.941)
US findings (II trimester)	0.125 (0.022–0.338)	0.796 (0.677–0.889)	0.154 (0.027–0.404)	0.754 (0.633–0.853)
US findings (III trimester)	0.235 (0.080–0.465)	0.863 (0.751–0.939)	0.364 (0.130–0.654)	0.772 (0.653–0.867)
MRI findings	NA	NA	NA	0.750 (0.631–0.848)

NA: Not available.

**Table 3:** Predictors of adverse outcome.

Neonatal Confirmed Infection		
Predictor	OR (95% CI)	*p*
GA at start of treatment	1.048 (0.939–1.170)	0.406
GA at end of treatment	1.232 (1.077–1.411)	0.002
Duration of treatment	1.135 (1.011–1.274)	0.032
Positive amniocentesis	32.857 (3.329–324.316)	0.003
Findings at echo	1.303 (0.123–13.774)	0.826
Interruption of treatment < 25 weeks	0.304 (0.097–0.953)	0.041
**Symptomatic Neonatal Infection**		
**Predictor**	**OR (95% CI)**	***p***
GA at start of treatment	0.919 (0.769–1.099)	0.353
GA at end of treatment	1.022 (0.862–1.212)	0.802
Duration of treatment	1.095 (0.931–1.288)	0.273
Positive amniocentesis	3.750 (0.331–42.467)	0.286
Echogenic bowel	1.000 (0.048–20.829)	1.000

GA: gestational age.

**Table 4:** Clinical Symptoms and Imaging Findings in Symptomatic CMV-Positive Infants.

Neonate	Prenatal US Findings	Prenatal MRI	Postnatal Neuroimaging Findings	Additional Findings
1	Normal	Np	Periventricular calcifications + vasculopathy (MRI)	Normal
2	Normal	Np	Periventricular calcifications + polymicrogyria (MRI)	Normal
3	Normal	Np	Periventricular calcifications + germinal matrix cysts (cUS)	Normal
4	Normal	Np	Normal	Abnormal transient otoacoustic emissions (TOAEs):
5	Normal	Np	Normal	Abnormal transient otoacoustic emissions (TOAEs):

Np = not performed.

## Data Availability

The datasets analyzed during the current study are available from the corresponding author upon request.

## Abbreviations

The following abbreviations are used in this manuscript:

cCMV	Congenital cytomegalovirus
ABR	Acoustic evoked potentials
MRI	Magnetic Resonance Imaging
VCV	Valaciclovir
